# Dugbe virus ovarian tumour domain interferes with ubiquitin/ISG15-regulated innate immune cell signalling

**DOI:** 10.1099/vir.0.048322-0

**Published:** 2013-02

**Authors:** S. Bakshi, B. Holzer, A. Bridgen, G. McMullan, D. G. Quinn, M. D. Baron

**Affiliations:** 1 School of Biomedical Sciences, University of Ulster, Cromore Road, Coleraine BT52 1SA, Northern Ireland; 2 Institute for Animal Health, Ash Road, Pirbright, Surrey GU24 0NF, UK

## Abstract

The ovarian tumour (OTU) domain of the nairovirus L protein has been shown to remove ubiquitin and interferon-stimulated gene 15 protein (ISG15) from host cell proteins, which is expected to have multiple effects on cell signalling pathways. We have confirmed that the OTU domain from the L protein of the apathogenic nairovirus Dugbe virus has deubiquitinating and deISGylating activity and shown that, when expressed in cells, it is highly effective at blocking the TNF-α/NF-κB and interferon/JAK/STAT signalling pathways even at low doses. Point mutations of the catalytic site of the OTU [C40A, H151A and a double mutant] both abolished the ability of the OTU domain to deubiquitinate and deISGylate proteins and greatly reduced its effect on cell signalling pathways, confirming that it is this enzymic activity that is responsible for blocking the two signalling pathways. Expression of the inactive mutants at high levels could still block signalling, suggesting that the viral OTU can still bind to its substrate even when mutated at its catalytic site. The nairovirus L protein is a very large protein that is normally confined to the cytoplasm, where the virus replicates. When the OTU domain was prevented from entering the nucleus by expressing it as part of the N-terminal 205 kDa of the viral L protein, it continued to block type I interferon signalling, but no longer blocked the TNF-α-induced activation of NF-κB.

## Introduction

Dugbe virus (DUGV) belongs to the genus *Nairovirus* of the family *Bunyaviridae*. It was isolated for the first time in Nigeria in 1964 from *Amblyomma variegatum* ticks (Causey, 1970) and was the first member of the genus *Nairovirus* to be characterized. It is considered to be endemic in arid regions and is one of the most commonly found tick-borne viruses in Africa, frequently isolated from ticks infesting market livestock (Burt *et al.*, 1996; David-West & Porterfield, 1974; Sang *et al.*, 2006). DUGV belongs to the Nairobi sheep disease serogroup, together with the highly pathogenic Nairobi sheep disease virus (NSDV) and the recently identified Kupe virus (Crabtree *et al.*, 2009). Although traditionally placed in different serogroups, DUGV also has high serological and genetic similarity to another pathogenic nairovirus, Crimean–Congo hemorrhagic fever virus (CCHFV). NSDV [known as Ganjam virus (GV) on the Indian subcontinent] causes haemorrhagic fever, gastroenteritis and abortion in sheep and goats with a mortality rate of up to 90 %, depending on the species, while CCHFV causes severe haemorrhagic fever in humans with a mortality rate of around 30 % (Ergönül, 2006; Marczinke & Nichol, 2002). DUGV itself is apathogenic in the livestock species in which it is found naturally, although it has been shown to cause thrombocytopaenia and a mild febrile illness in humans (Burt *et al.*, 1996) and is neuroinvasive in mice, killing both new born and adult mice (Boyd *et al.*, 2006; Coates & Sweet, 1990).

DUGV has a tri-segmented, negative-sense RNA genome. The three segments of the genome are designated S (small), M (medium) and L (large), encoding the nucleocapsid (N) protein, the glycoproteins (G_N_ and G_C_) and the L protein, respectively. The L proteins of nairoviruses have been found to be approximately twice the size of the L proteins of other members of the family, with the viral RNA-dependent RNA polymerase present at the C terminus and a so-called ovarian tumour-like protease (OTU) domain and a zinc finger domain at the amino terminus (Schmaljohn & Nichol, 2007).

Early induction of interferons (IFNs) is essential for controlling the replication of viruses in the host. The binding of IFN-α/β to its receptor activates the Janus kinase-signal transducer and activator of transcription (JAK/STAT) pathway, inducing the transcription of stimulated genes (ISGs), including IFN-α/β (Platanias, 2005). One of the most important ISGs expressed is ISG15, which is a 15 kDa ubiquitin-like protein with two ubiquitin-like domains, and is processed from a 17 kDa precursor (Blomstrom *et al.*, 1986; Farrell *et al.*, 1979; Haas *et al.*, 1987). It has been implicated in exerting a direct or indirect antiviral effect (reviewed by Harty *et al.*, 2009). It is primarily expressed upon the activation of the JAK/STAT pathway.

Ubiquitination is a reversible post-translational modification with a multitude of functions. Ubiquitin plays a very important role in the regulation of the innate immune response. Early IFN induction via RIG-I-like receptors (RLRs), Toll-like receptors/interleukin-1 receptor (TLRs/IL-1R) or the tumour necrosis factor α/nuclear factor-κB (TNF-α/NF-κB) pathway are regulated by polyubiquitination of a host of proteins through Lys48 or Lys63 of ubiquitin. The importance of ubiquitination is underscored by the fact that a host of cellular deubiquitinating enzymes downregulate these pathways (reviewed by Bhoj & Chen, 2009).

The N- and C-terminal domains of ISG15 share 29 and 31 % sequence similarity, respectively, with ubiquitin (Jeon *et al.*, 2010). Conjugation of ISG15 to its protein substrates follows the same pattern as ubiquitin, requiring UBE1L as the E1-activating enzyme and UbcH8 as the E2-conjugating enzyme, in humans Herc5 acts as the E3 ligase, while in mice mHerc6 performs this function. The mechanism responsible for the antiviral activity of ISG15 is not clearly understood. ISG15 has been found conjugated to proteins involved in direct or indirect antiviral activity including RIG-I, JAK1, STAT1, interferon regulatory factor 3 (IRF3) and protein kinase R (PKR) (Malakhov *et al.*, 2003; Zhao *et al.*, 2005). UBE1L^−/−^ mice exhibit increased susceptibility to influenza virus B (Lai *et al.*, 2009). Notably, ISG15^−/−^ mice have been shown to have increased susceptibility to influenza A, influenza B, herpes simplex virus 1, murine gamma herpes virus and Sindbis virus (SNV) (Lenschow *et al.*, 2007), while IFN-α receptor^−/−^ (IFNAR^−/−^) mice can be rescued from SNV infection by expression of ISG15, but not by expression of a mutated ISG15 that cannot be coupled to other proteins, suggesting that the conjugation of ISG15 to its cellular substrates is essential for it to exert its antiviral effect (Lenschow *et al.*, 2005). It has also been shown that ISGylation of IRF3 prevents its ubiquitination and degradation, enhancing its translocation to the nucleus, consequently, the relative amount of IRF3 is considerably reduced in ISG15^−/−^ cells compared with ISG15^+/+^ cells (Lu *et al.*, 2006). ISG15 negatively regulates important cell signalling pathways such as RLR signalling and activation of NF-κB (Kim *et al.*, 2008; Minakawa *et al.*, 2008). On the other hand, protein deISGylation negatively regulates the JAK/STAT pathway (Malakhova *et al.*, 2003).

Early IFN induction, type I IFN action and the production of cytokines such as TNF-α are essential for mounting an effective innate immune response against viral infection. Any strategy allowing viruses to inhibit or avoid the innate immune response will aid in the establishment of a potent viral infection. It has previously been shown that the isolated DUGV OTU domain has deubiquitinating and deISGylating activity (Frias-Staheli *et al.*, 2007), and we wished to investigate the implications of this for viral pathogenesis. Here, we show that, while the DUGV OTU domain inhibits both the TNF-α/NF-κB and JAK/STAT pathways, and abolishing the catalytic activity of the OTU domain resulted in a complete loss of both deubiquitinating and deISGylating activity and a reduction in the inhibition of the TNF-α/NF-κB and JAK/STAT pathways, a much larger OTU-containing protein that could not enter the nucleus no longer inhibited the TNF-α signalling pathway, while still blocking IFN signalling.

## Results

### DUGV OTU has deubiquitinating and deISGylating activity

**Fig. 1.**
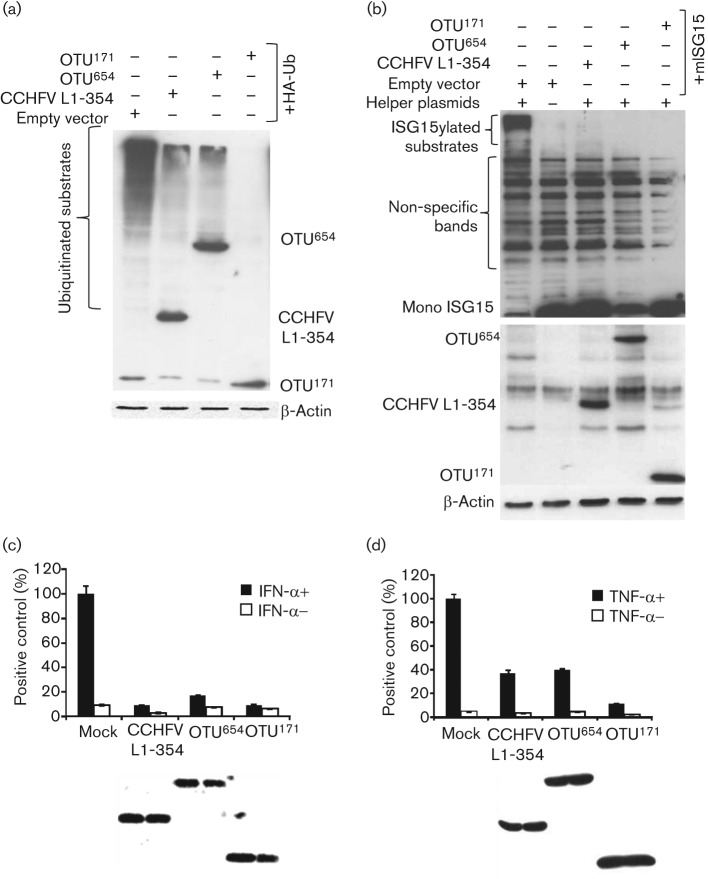
Effect of DUGV OTU^171^ and OTU^654^ on ubiquitination and ISGylation and on IFN and TNF-α signalling. (a) HEK293 cells were co-transfected with 500 ng of pcDNA3.1-HA-Ub and plasmids encoding OTU^171^ (500 ng), OTU^654^ (600 ng), CCHFV L1-354 (125 ng) or empty vector. Cells were harvested 30 h post-transfection in SDS-sample buffer and analysed by Western blot using HRP-conjugated rat anti-HA antibody. (b) Vero cells were co-transfected with His-mISG15 (250 ng), helper plasmids (250 ng each) and plasmids encoding OTU^171^ (400 ng), OTU^654^ (480 ng), CCHFV L1-354 (100 ng) or empty vector. Cell protein was harvested as in (a). ISGylated protein was detected using a mouse anti-6His antibody; OTU^171^/L expression was detected using HRP-conjugated rat anti-HA antibody. Actin was used as a loading control in (a) and (b). (c) Vero cells were co-transfected with 100 ng of pGL3-Mx1-luc and 200 ng of pJATLACZ along with plasmids encoding OTU^171^, OTU^654^, CCHFV L 1-354 or empty vector as in (a). Cells were treated with IFN-α (1000 U ml^−1^) 42 h post-transfection and harvested 6 h post-treatment. The ratio of luciferase and β-galactosidase activities was taken as the relative light units (RLUs). Results from three experiments were combined by setting the RLUs induced by IFN-α in the presence of empty vector to 100 %. Error bars show sd of the normalized data. (d) HEK293 cells were co-transfected with 250 ng of p6κB-luc and 200 ng of pJATLACZ along with plasmids encoding OTU^171^, OTU^654^, CCHFV L 1-354 or empty vector as in (a). Cells were treated with TNF-α (10 ng ml^−1^) 24 h post-transfection and harvested 6 h post-treatment. RLUs were determined as in (c). Results from three experiments were combined by setting the RLUs induced by TNF-α in the presence of empty vector to 100 %. Error bars show sd of the normalized data.

In order to confirm the enzymic activity of the DUGV OTU domain, and to further investigate the effects of downstream L sequences on this activity, we generated two forms of the OTU domain expressing either the first 171 (OTU^171^) or the first 654 (OTU^654^) aa. OTU^654^ extends the expressed protein from the OTU domain to the conserved nairovirus L protein zinc finger domain. A plasmid expressing the first 354 aa of the CCHFV L protein was used as a positive control, and the ability of these proteins to block/reverse ubiquitination of host cell proteins assayed by using a plasmid expressing HA-tagged ubiquitin (Frias-Staheli *et al.*, 2007). OTU^171^, OTU^654^ and the positive control contain HA tags and hence appear on the same blot as ubiquitin and its conjugates. As shown in Fig. 1(a) both constructs were enzymically active, with OTU^171^ slightly more effective than either OTU^654^ or the CCHFV construct.

We also compared the effects of the two expression constructs on ISG15 conjugation. ISG15-conjugated proteins were generated by transfecting cells with a plasmid expressing 6His-tagged ISG15 along with plasmids encoding the three murine activating/ligating proteins (‘helper’ plasmids) (Frias-Staheli *et al.*, 2007). These four plasmids were co-transfected with empty vector, OTU^171^, OTU^654^ or the positive control. A further control omitted the ‘helper’ plasmids to show that detectable ISGylation takes place only in the presence of these proteins. As shown in Fig. 1(b), all the OTU-containing constructs were equally effective at deISGylating proteins, showing that the DUGV L protein has an active OTU domain, similar to the pathogenic viruses CCHFV and NSDV. The reduced effect of the longer constructs, seen previously with the CCHFV L protein (Frias-Staheli *et al.*, 2007), could be due to a different structural conformation of downstream sequences which may partially inhibit the activity of the OTU domain.

### DUGV OTU inhibits the JAK/STAT and the TNF-α/NF-κB pathways

The induction of the JAK/STAT pathway by IFN-α/β is critical for the expression of IFN-stimulated antiviral genes. In order to test the effect of the DUGV OTU on this pathway, Vero cells were co-transfected with a reporter plasmid (pGL3-Mx-1-luc) carrying a luciferase gene under the control of a type I IFN-responsive promoter along with the DUGV or CCHFV OTU expression plasmids. The amounts of each plasmid transfected were adjusted so that approximately the same amount of each viral protein was expressed, as determined by Western blot. The cells were then treated with IFN-α to induce transcription from the type I IFN responsive promoter. As shown in Fig. 1(c), a more than 90 % reduction in induced luciferase expression was observed in the presence of the OTU^171^ or CCHFV L1-354 constructs, while a reduction of approximately 83 % was observed in the presence of OTU^654^. This minor difference in the effects of the OTU^171^ and OTU^654^ constructs, even though statistically significant, could be due to small differences in levels of expression of the two proteins that are not visible on the Western blot.

The induction of NF-κB by TNF-α is essential for the expression of genes involved in cell survival, the inflammatory response and the regulation of apoptosis (Tamatani *et al.*, 1999; Hayden *et al.*, 2006). To test the effect of the DUGV OTU on this pathway, HEK293 cells were co-transfected with an NF-κB-responsive reporter plasmid (p6κB-luc) along with the DUGV or CCHFV OTU expression plasmids. Transcription was induced by treating cells with TNF-α. As shown in Fig. 1(d), an approximate 90 % reduction in induction of luciferase expression was observed in the presence of OTU^171^, whereas OTU^654^ or CCHFV L1-354 reduced induction by only 60 %. The differences in the effects on induction observed in the presence of CCHFV L1-354 and OTU^654^ compared with OTU^171^ were statistically significant. These results suggest that a shorter segment of the DUGV L protein expressing the OTU domain is more effective at deubiquitinating host cell proteins and at inhibiting the TNF-α/NF-κB pathway.

### The effect of catalytic mutants of the OTU domain on ubiquitination, ISGylation and the JAK/STAT and TNF-α/NF-κB pathways

**Fig. 2.**
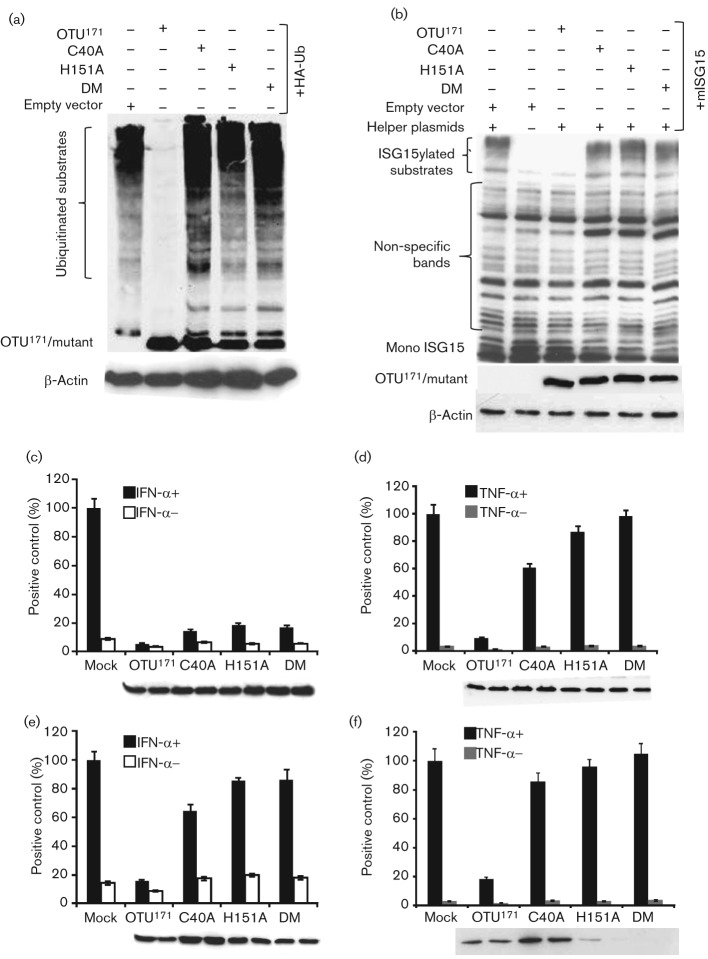
Effect of OTU mutants on activity in protein modification and cell signalling assays. (a) HEK293 cells were co-transfected with pHA-Ub and plasmids encoding OTU^171^, C40A, H151A, DM or empty vector. Cells were harvested and analysed by Western blot as Fig. 1(a). (b) Vero cells were co-transfected with pHis-mISG15, helper plasmids and plasmids encoding OTU^171^, C40A, H151A, DM or empty vector and harvested and analysed by Western blot as in Fig. 1(b). Actin was used as a loading control in (a) and (b). (c, e) Vero cells were co-transfected with 100 ng of pGL3-Mx1-luc and 200 ng of pJATLACZ along with (c) 500 ng or (e) 50 ng plasmid encoding OTU^171^, C40A, H151A or DM or empty vector. Cells were treated with IFN-α as in Fig. 1(c). Results from three experiments were combined by setting the RLUs induced by IFN-α in the presence of empty vector to 100 %. (d, f) HEK293 cells were co-transfected with 250 ng of p6κB-luc and 200 ng of pJATLACZ along with OTU^171^, C40A, H151, DM or empty vector at (d) 500 ng each or (e) 50 ng each. Cells were treated with TNF-α as in Fig. 1(d). Results from three experiments were combined by setting the RLUs induced by TNF-α in the presence of empty vector to 100 %. (c–f) Error bars show sd of the normalized data. The expression of wild-type and mutant OTU^171^ in a representative experiment is shown.

Based on previous studies on the CCHFV OTU domain which have shown that the cysteine and histidine residues at positions 40 and 151, respectively, form essential components of the catalytic site (Akutsu *et al.*, 2011; James *et al.*, 2011), we generated three point mutants of the DUGV OTU domain by mutating the cysteine (C40A) or histidine (H151A) or both (DM) to alanine. We then tested the effect of the different point mutants on the above-mentioned post-translational modifications and cell signalling pathways. As shown in Fig. 2(a) and (b), the introduction of these mutations into the DUGV OTU domain completely abolished its deubiquitinating and deISGylating activity, confirming that the catalytic activity of the DUGV OTU domain is responsible for deubiquitinating and deISGylating cellular proteins and that the cysteine and histidine residues at positions 40 and 151, respectively, are essential for this activity.

Interestingly, when tested in reporter gene assays, the C40A-mutated OTU still had a clearly measurable effect on the TNF-α/NF-κB pathway, while all three mutants were almost equally as effective as the OTU^171^ at blocking the JAK/STAT pathway (Fig. 2c, d). The differences in induction between C40A and the negative control for the TNF-α/NF-κB pathway, and between each of the three mutants and the negative control for the JAK/STAT pathway, were found to be statistically significant. The reporter gene assays were performed using 500 ng each of OTU^171^ and the three mutants, as this was the amount of plasmid used in our earlier studies, as well as in comparable published studies on other OTU^171^ (Frias-Staheli *et al.*, 2007; Holzer *et al.*, 2011). We hypothesized that the mutant OTU^171^, although lacking catalytic activity, may continue to bind to their substrates, and this binding might be more stable than normal as the substrate is not cleaved by the mutant OTU^171^. This binding might inhibit the activity of the target proteins somewhat if the OTU proteins were being expressed in large excess.

To test this hypothesis, the minimum amount of OTU^171^ required for blocking these pathways was determined. We found that a 10-fold lower amount of plasmid (50 ng) led to the expression of sufficient OTU^171^ to block most of the TNF-α- or IFN-α-induced expression of luciferase in our assays (not shown). We then compared the activity of similar amounts of mutant OTU^171^ in the reporter gene assays. As shown in Fig. 2(e), the mutant OTU^171^ had a much reduced effect on the JAK/STAT pathway relative to that of the wild-type protein when the amount of each expression plasmid was reduced to 50 ng. The reduction in induction in the presence of C40A and H151A, but not that in the presence of DM, was statistically significant.

When we assayed the effects of the catalytic site mutants on the TNF-α/NF-κB pathway using the reduced amount of expression plasmids, an apparent instability of the two proteins containing the H151A mutation in HEK293 cells became noticeable, and mutants H151A and DM were hardly detectable by Western blot. In contrast, the C40A mutant was expressed as well as, if not better than, the wild-type OTU^171^. Despite this, the C40A mutant no longer had a significant effect on TNF-α signalling.

### Effects of cellular distribution on OTU activity

**Fig. 3.**
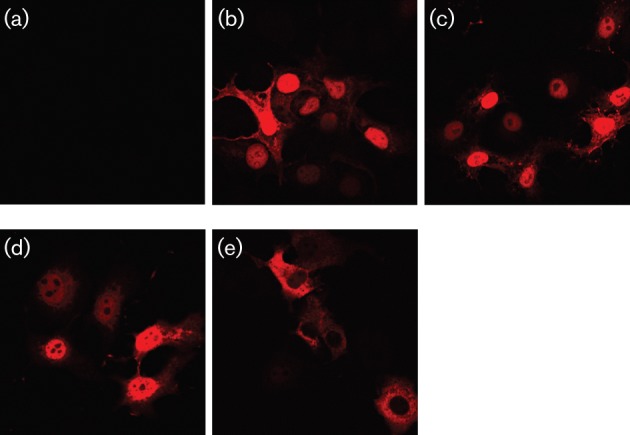
Cellular localization of OTU-containing constructs. Vero cells were transfected with (a) 1 µg of empty vector or 1 µg plasmid encoding (b) OTU^171^, (c) OTU^654^, (d) CCHFV L1-354 or (e) OTU++ and the cellular localization of the expressed protein visualized by immunofluorescence using Alexa Fluor568-coupled rat anti-HA.

In control experiments to look at the expression of the various constructs in transfected cells using immunofluorescence, we observed that OTU^171^, OTU^654^ and the CCHFV OTU construct all concentrated in the nuclei of cells in which they were expressed, suggesting that the proteins either have a cryptic or non-canonical nuclear localization signal (NLS) or are retained in the nucleus, perhaps by interaction with one or more nuclear proteins, after diffusion in through nuclear pores. We constructed an extended form of the DUGV OTU (OTU^1795^), consisting of the first 1795 aa of L. This protein, with a predicted size of 207 kDa, is too large to passively pass through the nuclear pore, and immunofluorescence showed that it was excluded from the nuclei of cells in which it was expressed (Fig. 3), suggesting there is no NLS. The retention of this protein in the cytoplasm agrees with similar results we have seen with the first half of the L protein of NSDV/GV, or with the whole NSDV/GV L protein in virus-infected cells (unpublished), as well as the localization of the CCHFV L protein when expressed on its own (Bergeron *et al.*, 2010).

**Fig. 4.**
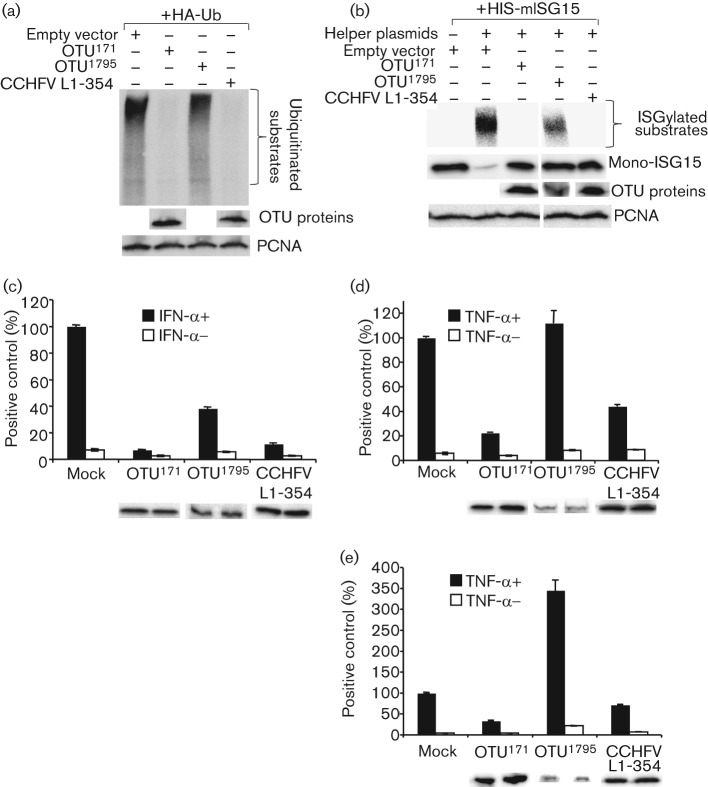
Activity of OTU^1795^ in protein modification and cell signalling assays. (a) HEK293 cells were co-transfected with 350 ng of pHA-Ub and plasmids encoding OTU^171^ (50 ng), OTU^1795^ (500 ng) or CCHFV L1-354 (65 ng), or empty vector. Cells were harvested 30 h post-transfection in SDS-sample buffer. The blot was probed using an HRP-conjugated rat anti-HA antibody. (b) Vero cells were co-transfected with pHis-mISG15 (250 ng), helper plasmids (250 ng each) and plasmids encoding OTU^171^, OTU^1795^ or CCHFV L1-354, or empty vector as in (a). Cells were harvested as in (a). The blot was probed using a mouse anti-6His antibody. OTU protein expression was detected in separate blots using HRP-conjugated rat anti-HA antibody. PCNA was used as a loading control in (a) and (b). (c) Vero cells were co-transfected with 100 ng pGL3-Mx1-luc and 200 ng pJATLACZ along with plasmids encoding OTU^171^ (60 ng), OTU^1795^ (1000 ng) or CCHFV L 1-354 (65 ng), or empty vector. Cells were treated with IFN-α and RLUs determined as in Fig. 1. (d, e) HEK293 cells were co-transfected with 250 ng p6κB-luc and 200 ng pJATLACZ along with plasmids encoding OTU^171^ (50 ng), OTU^1795^ [(d) 500 ng, (e) 1000 ng)] or CCHFV L 1-354 (65 ng), or empty vector. Cells were treated with TNF-α and RLUs were determined as in Fig. 1. (c–e) Error bars show sd of the normalized data from three experiments. Expression of OTU-containing protein from a representative experiment, as determined by Western blot, is shown.

The OTU^1795^ protein, probably because of its large size, was expressed relatively poorly in transfected cells, and there was always much less of this protein than the OTU^171^ or CCHFV controls, despite the large difference in amounts of each plasmid used (50 ng of OTU^171^ plasmid vs 500 ng of OTU^1795^ plasmid). The OTU element in OTU^1795^ was clearly still functional, as seen by the reduced amount of ubiquitinated proteins seen when this plasmid was co-transfected with the HA-Ub construct (Fig. 4a) (Note that it is impossible to see the HA-tagged OTU^1795^ against the background of high molecular mass HA-Ub proteins in the deubiquitination assays). Similarly, inclusion of OTU++ in the ISGylation assay showed a reduced level of ISG15 incorporation into protein, and a concomitant increased amount of free ISG15 in transfected cells. (Fig. 4b). In the reporter gene assays, however, a clear difference was seen between the activity of the OTU^1795^ protein in the IFN and TNF-α signalling assays. The OTU^1795^ protein, despite its lower expression level, was still effective at blocking type I IFN signalling (Fig. 4c), but was no longer active in blocking TNF-α activation of NF-κB (Fig. 4d). When the amount of OTU^1795^ plasmid was increased even more, to see if the amount of this protein was the limiting factor, we actually found that the activity of TNF-α in this assay was magnified (Fig. 4e).

## Discussion

The dual deubiquitinating and deISGylating potential of DUGV OTU and other nairoviral OTU^171^ is in contrast with the simple deubiquitinating activity of mammalian OTU domain-containing proteins such as A20 and Cezanne, which have specific targets for deubiquitination and have no deISGylating activity (Enesa *et al.*, 2008; Wertz *et al.*, 2004). Until now only one ISG15-specific protease (UBP43) has been identified in the human proteome (Malakhov *et al.*, 2002). As a consequence of having an apparent global effect on cellular ubiquitination and ISGylation, the nairoviral OTU^171^ will be able to interfere with multiple cell signalling pathways and control mechanisms regulated by ubiquitin and ISG15. DUGV is closely related to the highly pathogenic nairoviruses NDSV/GV and CCHFV, but is substantially less pathogenic than these viruses in the hosts with which it is naturally associated. In the absence of a wild-type isolate of DUGV that would grow in mammalian cells, we have used expression plasmids to investigate whether the DUGV OTU confers the ability to control innate immune signalling in the same way as those of NSDV/GV and CCHFV (Frias-Staheli *et al.*, 2007; Holzer *et al.*, 2011). We observed that the DUGV OTU was highly effective at deubiquitinating and deISGylating cell proteins, confirming previous observations (Frias-Staheli *et al.*, 2007). We showed that the DUGV OTU was able to block the type I IFN (JAK/STAT) signalling pathway (as previously shown for the NSDV/GV OTU; Holzer *et al.*, 2011) and that of TNF-α/NF-κB (as previously shown for the CCHFV OTU; Frias-Staheli *et al.*, 2007). The introduction of mutations into the DUGV OTU domain catalytic site, as predicted from comparison with the structure of the CCHFV OTU (Akutsu *et al.*, 2011; James *et al.*, 2011), completely abolished its deubiquitinating and deISGylating activity, confirming its role in deubiquitination and deISGylation and the role of C40 and H151 in its catalytic activity.

The OTU mutants revealed interesting differences in the interaction of the DUGV OTU with the two signalling pathways. When an excess of catalytically inactive mutant was used for the reporter gene assays, induction of the JAK/STAT pathway was still reduced by more than 80 %, and even when the amount of plasmid encoding the wild-type or mutant OTU^171^ was reduced to 50 ng per transfection, the C40A mutant continued to show a significant effect on induction of the JAK/STAT pathway (by approximately 33 %), suggesting that the targets in this case are bound with fairly high affinity by the OTU, even if it is not cleaving the substrate. We did not observe this residual activity in similar mutants of the NSDV/GV OTU (Holzer *et al.*, 2011), but this was largely because those studies were carried out with pcDNA-based constructs, which give >fivefold lower expression of the respective protein. The C40A mutant had a detectable effect on the TNF-α/NF-κB signalling pathway when the mutant protein was in excess, but had no significant effect at limiting doses of OTU. These observations suggest that the binding ability of the OTU domain is sufficient for it to exert an effect, and the affinity of the OTU^171^/mutants is higher for the target host cell protein involved in the IFN action pathway than that involved in the TNF-α signalling pathway, providing further evidence that the nairovirus OTU^171^ are acting through multiple specific targets in the host cell (Holzer *et al.*, 2011). Further studies are planned to identify host cell proteins bound by the virus OTU using catalytic site mutants, though it is clear that care must be taken over the mutant or cell line used, given the obervation that the H151A mutation appears to destabilize the protein in HEK293s (e.g. Fig. 2f; similar observations were made during ubiquitination assays), but not in Vero cells. It is possible that the HEK293 cells have a higher level of proteasomal activity, and that the H151A mutation has a stronger effect on folding of the OTU protein than the C40A mutation, leading to a more rapid turnover of proteins containing the former mutation. This more rapid turnover only became apparent at low levels of protein expression.

The effect of the DUGV OTU domain on innate immune cell signalling is similar to that observed for CCHFV and NSDV/GV OTU domains. The CCHFV OTU domain has been shown to inhibit the TNF-α/NF-κB pathway and interfere with RLR-mediated signalling (Frias-Staheli *et al.*, 2007; van Kasteren *et al.*, 2012). The NSDV/GV OTU, as well as the whole virus, has been shown to interfere with type I IFN induction and both type I and II IFN signalling (Holzer *et al.*, 2011). One potential problem with interpreting these studies is that assessment of the activity of these viral proteins has mostly been carried out on small proteins or parts of proteins, not least because of the difficulty of expressing the full nairovirus L protein (460 kDa) from a plasmid. We observed that the relatively small OTU^171^, OTU^654^ and CCHFV L1-354 were all concentrated in the nuclei of transfected cells, and it was possible that the effects observed with the virus OTU domain could be partially due to its access to the nucleus, where the viral L protein is not normally found. Indeed we found that a protein corresponding to the N-terminal 45 % of the DUGV L, which did not enter the nucleus, had no effect on TNF-α activation of NF-κB, while its OTU is still enzymically active and the protein still blocked type I IFN signalling. This suggests that the observed blockade of TNF-α signalling by the CCHFV L1-169 (Frias-Staheli *et al.*, 2007) is possibly an artefact, and indeed we found that expression of sufficient cytosol-located DUGV OTU led to an increased, not decreased, response of the cells to TNF. On the other hand, the potentiation of virus pathogenesis observed with the CCHFV OTU is likely correct, since that was carried out with a larger part of the whole L protein (CCHFV L1-1325), which would be too large to enter the nucleus passively. It is clearly important to confirm results using OTU-containing constructs that are too large to freely enter the nucleus, such as the N-terminal half of the NSDV/GV L protein (Holzer *et al.*, 2011) or otherwise as near the normal state as possible, such as the complete nsp2-3 protein of equine arterivirus (van Kasteren *et al.*, 2012).

The expressed DUGV OTU domain is highly effective at interfering with IFN signalling. Despite this, DUGV is still relatively apathogenic compared with CCHFV and NSDV. A DUGV isolate adapted to mammalian cell culture induced a 30–50-fold higher cytokine/chemokine response in macrophages infected *in vitro*, compared with CCHFV (Peyrefitte *et al.*, 2010), although recent observations on the accumulation of DI particles during cell culture passage of an RNA virus (Killip *et al.*, 2011) make it important to confirm the absence of such contaminants in studies on the whole virus. It is also possible that a combination of mechanisms may be required in order to effectively evade the innate immune response. CCHFV shows delayed type I IFN induction and generates 5′ monophosphate-containing ssRNA molecules during replication, allowing it to escape detection by RIG-I (Andersson *et al.*, 2008; Habjan *et al.*, 2008). NSDV/GV also shows a delay in type I IFN production (Holzer *et al.*, 2011). It is possible that these two viruses combine the effects of the OTU domain and their potential to delay type I IFN production in order to evade the innate immune response.

## Methods

### 

#### Cells and viruses.

The human embryonic kidney (HEK293) cell line was obtained from Dr Paul Thompson, University of Ulster, Coleraine. Vero cells (African green monkey kidney cells) used were a derivative expressing the canine Signalling Lymphocyte Activation Molecule (SLAM) and were obtained from Dr Paul Duprex of Queen’s University, Belfast, UK. Cells were maintained in high glucose Dulbecco’s modified Eagle’s medium supplemented with 10 % FBS, penicillin (100 U ml^−1^) and streptomycin sulphate (100 µg ml^−1^). Dugbe virus (strain ArD44313) was originally obtained from Dr Ernie Gould, NERC Centre for Ecology and Hydrology, Oxford and grown in suckling mice brain prior to passage in BHK cells. Titration of virus and RNA extraction was carried out as described previously (Bridgen *et al.*, 2002).

#### Plasmids.

Plasmid pJATLACZ was the kind gift of Professor Steve Goodbourn, St. George’s Hospital Medical School, London, UK. pGL3-Mx1P-luc was the kind gift of Professor Georg Kochs, Department of Virology, University of Freiburg, Germany. Plasmid p6κB-luc (in which luciferase is expressed under the control of six copies of the NF-κB-responsive element from human immunodeficiency virus 1) was provided by Dr Julian Seago, IAH, UK. Plasmids for ubiquitination experiments (pcDNA3.1-HA-Ub, pCAGGS-MCSII-HA-CCHFV-L1-354) were kind gifts of Dr Natalia Frias-Staheli, Mount Sinai School of Medicine, New York, USA. Plasmids for ISGylation experiments (pCAGGS-MCS-6HismISG15, plasmids expressing mUBE1L, Ubcm8 and mHerc6) were kindly provided by Professor Deborah Lenschow, Washington University, USA. Plasmid pCAGGS-MCSII was the gift of Professor Adolfo Garcia-Sastre, Mount Sinai School of Medicine, New York, USA.

Clones representing the entire DUGV L segment (strain ArD44313) were previously constructed under control of the T7 promoter in pBluescript SK+. Nucleotides 1–500 were amplified from cDNA using *Pfu* DNA polymerase and primers L1 (5′-TCTCAAAGACATCAATCCCC-3′) and L62 (5′-GCTGTTCTATTATCCTAAGC-3′) and cloned into the *Eco*RV site of pBluescript SK+. This was extended to nt 5720 by insertion of an *Nsi*I–*Bam*HI fragment from plasmid DEGDUGL, a kind gift of Professor Pat Nuttall, NERC Centre for Ecology and Hydrology, Oxford, UK (A. Bridgen, unpublished results). This clone, pT7-5′DEGL, was subcloned into plasmid pTM1 then used as a PCR template to amplify the regions coding for the first 171 and 654 aa of the L protein using the KOD Hot start DNA polymerase (Novagen). These two regions were cloned into pCAGGS-MCSII with N-terminal HA-tags. The catalytic site point mutations were introduced by overlap PCR mutagenesis into the L1-171 expression construct. The correct ORFs of all PCR products were confirmed by sequencing. To create OTU^1795^, the 4.9 kb *Nsi*I–*Xho*I fragment from pTM1-5′DEGL was inserted into pCAGGS-OTU^171^ cut with the same enzymes. The resultant plasmid expresses aa 1–1795 of DUGV L, preceded by an HA tag. Large-scale plasmid preparations were performed by either CsCl gradient purification or using the Plasmid Maxi kit (Qiagen).

#### Antibodies.

Mouse mAb anti-His was purchased from Sigma Aldrich, HRP-conjugated rat anti-HA antibody was purchased from Roche and rabbit anti-β-actin antibody was purchased from NEB. Mouse anti-PCNA antibody was purchased from Santa Cruz. HRP-conjugated anti-mouse antibody was purchased from GE Healthcare and HRP-conjugated anti-rabbit antibody was purchased from Sigma Aldrich.

#### Transfections, immunoblotting and reporter gene assays.

Transfections were carried out using the transfection reagent TransIT LTI (Mirus) according to the manufacturer’s instructions. Three microlitres of TransIT was used per μg of plasmid DNA. The assays of ubiquitination, ISGylation and the reporter gene assays were carried out in either Vero or HEK293 cells, depending on which gave the clearest result for a particular combination of plasmids and/or antibodies. HEK293 cells were plated at 5×10^5^ per well in 12-well plates and transfected the next day, whereas Vero cells were plated at 8×10^4^ per well in 12-well plates and transfected after 6 h. For immunoblotting, cells were harvested using 100 µl SDS-sample buffer (Cell Signalling Technology) containing a protease inhibitor cocktail (1 : 100; Sigma Aldrich). SDS-PAGE and immunoblotting were performed as described previously (Nanda & Baron, 2006). Luciferase and β-galactosidase readings were taken as described previously (Holzer *et al.*, 2011). The luminescence and absorbance readings were taken using a Fluostar Omega and MARS Analysis software (BMG Labtech). In each experiment, the total amount of plasmid transfected in each well was kept constant by adding empty vector. The reporter assay results were statistically analysed using one-way ANOVA. The post-hoc analysis was performed using Dunnett’s test (*P* = 0.05) for the multiple conditions in the assay (SPSS; IBM).

#### Immunofluorescence staining and confocal microscopy.

Vero cells were grown on coverslips to approximately 50 % confluence and transfected with 1 µg of plasmid DNA. One day post-transfection, the cells were fixed with 3 % paraformaldehyde and permeabilized with 0.2 % Triton X-100 at room temperature. The fixed and permeabilized cells were blocked with 5 % goat serum for at least 30 min before processing for antibody staining. Initial confocal microscope images were obtained using the manufacturer’s software (Leica Confocal Software). All experiments were performed at least three times, and representative images are shown.
